# Knowledge of the statutory fertility provision and its associated factors among reproductive-aged women: a cross-sectional study conducted in Shandong Province, China

**DOI:** 10.3389/fpubh.2026.1871125

**Published:** 2026-07-27

**Authors:** Hongyan Hao, Lisha Wang, Jiaheng Zuo, Mengjun Cao, Zihan Zhou, CanXin Dai, HuiYu Shan, Na Sun, Qiang Wang

**Affiliations:** 1 College of Public Health, Shandong Second Medical University, Shandong, China; 2 Department of Human Resources, Dezhou Center for Disease Control and Prevention, Dezhou, China; 3 Primary Health Section, Health Commission of Dezhou, Dezhou, China; 4 Department of Biostatistics, Shandong Second Medical University, Shandong, China; 5 Department of Epidemiology, Shandong Second Medical University, Shandong, China

**Keywords:** associated factors, cross-sectional survey, knowledge of the statutory fertility provision, logistic regression, reproductive-aged women

## Abstract

**Objective:**

Knowledge of the statutory fertility provision is a critical but understudied determinant of effective policy implementation in the context of China’s declining fertility rate. This study aimed to identify factors associated with such knowledge among reproductive-aged women aged 18–45 in Shandong Province, so as to provide empirical evidence for optimizing the fertility policy publicity system.

**Methods:**

A cross-sectional survey was conducted using multistage stratified cluster sampling among reproductive-aged women in Shandong Province. Data were collected using a researcher-developed, self-administered questionnaire. The chi-square test and multivariate logistic regression were used to identify factors associated with knowledge of the statutory fertility provision.

**Results:**

A total of 2,422 valid questionnaires were included. Only 43.31% of participants had adequate knowledge of the statutory fertility provision. Multivariate logistic regression identified four key factors independently associated with knowledge: residence, monthly total family expenditure, expected age at first birth, and preferred number of children (all *p* < 0.05). Rural residents had significantly lower knowledge than urban residents. Higher knowledge was observed among women with moderate monthly expenditure (4001–8,000 Yuan), those planning first birth at 28–34 years, and those expecting 3 or more children.

**Conclusion:**

Knowledge of the statutory fertility provision among reproductive-aged women in Shandong Province is low and associated with multiple sociodemographic and fertility-related factors. Targeted policy communication strategies that address rural–urban disparities and prioritize key groups are needed to improve policy awareness.

## 1 Introduction

Long-term balanced population development is the core support for sustainable economic and social progress (1). At present, China’s population growth has entered a new stage of negative growth. In 2024, the total number of births nationwide was 9.54 million, with a natural growth rate of −0.99‰ (2). From the perspective of population age structure, China has entered a society with sub-replacement fertility and population aging (3). Low fertility and population aging have become major strategic issues affecting national development (4). To actively respond to this situation, China has successively introduced the two-child policy in 2016 and three-child policy in 2021, as well as their supporting measures (5). By providing a series of fertility policies including fertility subsidies, leave protection, and childcare services, China is building a fertility-friendly society to facilitate the translation of fertility intention into actual fertility behavior (6, 7). Although a series of measures have been enacted, their effects are evident only in the short term, while long-term outcomes remain inadequate (8). Therefore, further analysis is required to examine the reasons why fertility policies have failed to achieve sustained effectiveness in stimulating childbirth.

When examining the reasons why the effects of fertility policies have failed to persist, apart from macro-level factors such as economic pressure and childcare costs, a fundamental and critical antecedent cannot be overlooked: the level of fertility policy awareness among the reproductive-aged population (9). Fertility policy awareness serves as the “first gateway” connecting policy supply to fertility behavior (10). In essence, it represents individuals’ subjective understanding of policy content, eligibility criteria, and potential benefits (11). Insufficient awareness directly hinders the realization of policy dividends and prevents support measures from being effectively translated into fertility incentives (12). Currently, fertility policy awareness in China suffers from three prominent issues: low levels of public knowledge, incomplete understanding, and inconvenient access to information (13–16). These problems are particularly pronounced among rural populations, individuals with lower educational attainment, and migrant groups (17). These issues have become salient weaknesses constraining the effectiveness of fertility policies. Notably, although scholarly attention to fertility support policies has increased, most research remains focused on analyzing the determinants of fertility intentions, while systematic, large-sample investigations specifically targeting fertility policy awareness relatively limited (18–22). Consequently, fundamental questions regarding the current level of awareness about fertility policy and its influencing factors remain to be fully elucidated.

To address this research gap, it is particularly necessary to conduct regional, large-sample, and multi-factor empirical research. In China, Shandong Province has a large population and significant urban–rural disparities (23). Therefore, the research findings on awareness of fertility policies among reproductive-aged women in Shandong Province may hold significant referential value for the nation as a whole (10). On this basis, this study took reproductive-aged women in Shandong Province as the research subjects to investigate the current level of knowledge of the statutory fertility provision and explore factors associated with it. The results may provide a reliable basis for optimizing fertility policy dissemination and improving the precision of policy communication, offering considerable practical significance and application value.

## 2 Materials and methods

### 2.1 Definition of participants

This cross-sectional study was conducted in Shandong Province in 2024, with reproductive-aged women aged 18–45 and holding Shandong household registration as the research subjects. From an international perspective, the definition of reproductive-aged women covers all women aged 15–49, regardless of marital status (24). However, the legal age of adulthood in China is 18 years, so this study set the minimum age at 18 years to ensure that all subjects were adults (25). Women over 45 years old experience a significant decline in fertility, ovarian function decline, and a substantial increase in delivery risks (26). Previous studies have also confirmed that women aged 40–49 make a low contribution to China’s fertility rate (27). Based on this, this study set the upper age limit at 45 years. To ensure sample representativeness and inter-group comparability, this study only included women with local household registration and excluded the floating population.

The inclusion criteria: ① Reproductive-aged women aged 18–45 residing in Shandong Province; ② Reproductive-aged women holding Shandong household registration; ③ Reproductive-aged women voluntarily participating in the study and being able to independently complete the questionnaire. Exclusion criteria: Women who had not resided in Shandong Province for more than 6 months prior to the survey. No other exclusion criteria were applied.

### 2.2 Sample size

In this study, the required sample size was calculated using the standard formula for estimating a single proportion (28).


$$n=\frac{Z_{\alpha /2}^{2}*p*(1-p)}{d^{2}}$$

In this formula, n is the required sample size, Z_α/2_ is the standard normal value corresponding to the desired confidence level, *p* is the estimated proportion and *d* is the allowable margin of error. A 95% confidence level was adopted (Z_α/2_ = 1.96). Based on the conservative estimate of the proportion related to knowledge of the statutory fertility provision, *p* = 0.5 was used to maximize the required sample size. The allowable margin of error (*d*) was set at 0.03. Substituting these values into the formula yielded:


$$n=\frac{1.96^{2}\times 0.5\times (1-0.5)}{0.03^{2}}\approx 1,067$$

The preliminary calculation indicated that the required sample size was 1,067. Considering the design effect due to multistage sampling (Design Effect, DEFF = 2) and an anticipated 10% rate of non-response rate, the final target sample size was calculated as follows (29).


$$n_{1}=n\times \text{DEFF}$$

In this formula, n is the preliminary sample size and DEFF is the design effect. Then, accounting for the expected non-response rate r, the final target sample size was calculated as follows.


$$n_{\text{final}}=\frac{n_{1}}{1-r}$$

Substituting the values from this study (*n* = 1,067, DEFF = 2, *r* = 0.10) gives:


$$n_{1}=1,067\times 2=2,134$$

$$n_{\text{final}}=\frac{2,134}{1-0.10}=2,371$$

Thus, the final target sample size was 2,371.

In this study, a total of 2,700 questionnaires were distributed. After excluding those with logical inconsistencies, missing data, or non-compliance with the inclusion criteria, 2,422 valid questionnaires were obtained, meeting the analysis requirements.

### 2.3 Sampling method

A stratified multistage cluster sampling design was employed in Shandong Province in 2024. The 16 cities in Shandong Province vary substantially in socioeconomic and demographic characteristics. First, the 16 cities were stratified into three strata according to economic development levels (e.g., per capita GDP), educational attainment, and geographic location. Second, three cities were randomly selected from each strata, yielding a total of nine sample cities. Third, one county-level city or district was randomly chosen from each of the nine sample cities, resulting in nine county- or district-level units. Fourth, one sub-district and one township were further randomly selected from each selected county- or district-level unit, resulting in nine sub-districts and nine townships. Fifth, one urban community and one administrative village were randomly drawn from each sub-district and township unit, respectively, yielding 18 final survey sites (9 urban communities and 9 administrative villages). Eligible reproductive-aged women who met all inclusion criteria were invited to complete the survey independently either during their visits to local healthcare centers or via community residential rosters. A target sample size of at least 140 eligible women was set for each final survey site; if the number of eligible women in a selected site was below this threshold, an additional community or village within the same sub-district or township was randomly selected as a supplementary site. At each stage, the number of urban communities and rural villages was proportionally allocated according to the actual number of reproductive-aged women in each area, ensuring balanced urban–rural representation and overall sample representativeness.

A total of 2,700 respondents were recruited, of which 2,422 valid questionnaires were retained for analysis.

### 2.4 Questionnaire survey

The questionnaire used in this study was developed by the research team based on a comprehensive literature review of fertility policy awareness and related factors. The initial draft was reviewed and refined by a panel of five experts in public health and demography to ensure content validity. The final questionnaire was self-administered, with all participants completing it independently without interviewer assistance. Research assistants were available on-site to address any questions regarding questionnaire comprehension.

The questionnaire included five categories of multi-dimensional variables: demographic characteristics (e.g., age, place of residence, educational level, marital status, type of original family, occupation), economic status (monthly household income and expenditure), traditional fertility concepts (degree of influence), fertility intention (related indicators), expectations from people around them (impact of external expectations). The selection of research variables was guided by the Theory of Planned Behavior (TPB) (30). While TPB is traditionally designed to predict behavioral intentions, this study extends its framework to explore factors linked to respondents’ policy information acquisition—specifically, dimensions that may reflect individuals’ willingness to seek out and comprehend fertility policy information. The analytical framework of behavioral attitude, subjective norm, and perceived behavioral control was extended to the formation process of policy awareness. Accordingly, the variables associated with knowledge of the statutory fertility provision were divided into three dimensions. The first is the cognitive attitude dimension, which includes indicators such as traditional fertility concepts and fertility intention. This dimension reflects the relationship between the research subjects’ subjective fertility tendencies and their willingness to acquire policy knowledge. The second is the social normative dimension, which covers the type of original family and the expectations from society and people around them. This dimension embodies the role of the external environment in motivating the research subjects and shaping their access to policy information. The third is the socioeconomic-cognitive dimension, which comprises household economic status, occupation, educational level, and other related factors. These factors comprehensively characterize the research subjects’ capabilities and resource conditions for acquiring and understanding fertility policies.

Based on the TPB framework, the following hypotheses were formulated: H1: The cognitive attitude dimension (traditional fertility concepts and fertility intention) is positively associated with knowledge of the statutory fertility provision. H2: The social normative dimension (family of origin and social expectations) is positively associated with knowledge of the statutory fertility provision. H3: The socioeconomic-cognitive dimension (household economic status, occupation, and educational level) is positively associated with knowledge of the statutory fertility provision.

In addition, the questionnaire included an outcome variable measuring knowledge of the statutory fertility provision. The outcome variable was defined as participants’ knowledge of the statutory fertility provision under China’s current three-child policy. It was measured by a single questionnaire item: “According to the current national fertility policy, what is the maximum number of children a couple is allowed to have?” Respondents who selected the correct answer (“three children”) were classified as having clear knowledge of the statutory fertility provision, while those who selected an incorrect answer or responded “unsure” were classified as having limited knowledge. That this single-item measure captures only the most fundamental aspect of fertility policy cognition—knowledge of the basic legal rule—and does not reflect awareness of other policy dimensions such as maternity leave, childcare subsidies, eligibility criteria, or policy access procedures.

Relevant items of the questionnaire were designed with a 5-point Likert scale, and the influence degree of each factor was quantified by scores from 1 to 5 (1 = No influence, 2 = Little influence, 3 = Moderate influence, 4 = Great influence, 5 = Extremely great influence). A higher score indicated a stronger association between the factor and knowledge of the statutory fertility provision, and the details of items and their scoring assignment are shown in Table 1. Prior to the formal survey, a pilot survey was conducted among 200 reproductive-aged women aged 18–45 to revise the items and improve the questionnaire design. The reliability and validity test of the formal survey showed that the Kaiser-Meyer-Olkin (KMO) value was 0.940, Bartlett’s test of Sphericity was significant (*p* < 0.001), and Cronbach’s alpha coefficient reached 0.955. These results indicated that the questionnaire had good reliability and validity, and could be used for data collection and analysis in this study.

**Table 1:** Univariate analysis of factors associated with knowledge of the statutory fertility provision.

Variables	Knowledge of the statutory fertility provision n(%)		χ^2^	*p*
	Vague	Clear		
Residence(Urban/Rural)	960(53.93)/413(64.33)	820(46.07)/229(35.67)	20.776	<0.001
Age/Years Old(18–27/28–37/38–45)	294(46.96)/583(56.71)/496(64.58)	332(53.04)/445(43.29)/272(35.42)	43.602	<0.001
Educational background(junior high school or below/high school or vocational/college/postgraduate or above)	154(77.00)/210(68.85)/ 892(53.16)/117(48.95)	46(23.00)/95(31.15)/ 786(46.84)/122(51.05)	66.326	<0.001
Marital status(unmarried and childless/married and childless/married and childbearing /widowed or divorced)	260(44.29)/115(55.83)/ 989(61.54)/9(40.91)	327(55.71)/91(44.17)/ 618(38.46)/13(59.09)	54.453	<0.001
Original family(nuclear family/stem family/joint family/single-parent family/reconstituted family)	646(53.26)/605(60.93)/ 76(61.79)/21(46.67)/25(52.08)	567(46.74)/388(39.07)/ 47(38.21)/24(53.33)/23(47.92)	16.642	0.002
Occupation(public sector or institutions/private or self-employed /manual laboror agriculture/flexible employment/ unemployed)	638(54.25)/151(64.81)/ 161(66.53)/362(53.08)/61(68.54)	538(45.75)/82(35.19)/ 81(33.47)/320(46.92)/28(31.46)	27.352	<0.001
Monthly total family income/yuan (Below 4,000/4001–8,000/8001-15000/Above 15,000)	260(68.24)/499(59.55)/ 423(51.27)/191(50.53)	121(31.76)/339(40.45)/ 402(48.73)/187(49.47)	39.196	<0.001
Monthly total family expenditure/yuan (Below 4,000/4001–8,000/8001-15000/Above 15,000)	583(62.69)/546(52.60)/ 215(53.75)/29(53.70)	347(37.31)/492(47.40)/ 185(46.25)/25(46.30)	22.300	<0.001
Influence of the concept of “continuing the family line”^†^ (1/2/3/4/5)	699(53.40)/249(61.18)/ 246(56.81)/76(64.41)/103(66.45)	610(46.60)/158(38.82)/ 187(43.19)/42(35.59)/52(33.55)	17.993	0.001
Influence of the concept of “more sons, more happiness”^†^ (1/2/3/4/5)	687(53.76)/234(59.09)/ 271(58.03)/81(63.28)/100(65.36)	591(46.24)/162(40.91)/ 196(41.97)/47(36.72)/53(34.64)	12.701	0.013
Influence of the concept of “raising sons for old age security”^†^ (1/2/3/4/5)	619(54.39)/240(57.69)/ 298(56.23)/105(62.50)/111(65.29)	519(45.61)/176(42.31)/ 232(43.77)/63(37.50)/59(34.71)	10.096	0.039
Expected age at first birth/years old (20–23/24–27/28–30/31-34/Above 35)	130(73.86)/678(61.64)/ 337(50.37)/75(43.10)/13(54.17)	46(26.14)/422(38.36)/ 332(49.63)/99(56.90)/11(45.83)	55.767	<0.001
Post-marital fertility time preference (≤1 Year/1–2 Years/3–4 Years/≥5 Years /Undecided)	61(67.78)/807(59.12)/ 170(50.60)/22(64.71)/173(54.40)	29(32.22)/558(40.88)/ 166(49.40)/12(35.29)/145(45.60)	13.886	0.008
Preferred number of children (0/1/2/3/More Than 3)	140(50.18)/387(55.92)/ 752(60.50)/52(40.00)/42(53.85)	139(49.82)/305(44.08)/ 491(39.50)/78(60.00)/36(46.15)	27.332	<0.001
Actual-scenario fertility expectation (0/1/2/3/More Than 3)	197(53.10)/558(53.76)/ 582(62.38)/29(43.28)/7(53.85)	174(46.90)/480(46.24)/ 351(37.62)/38(56.72)/6(46.15)	22.832	<0.001
Social expectation-fertility influence^†^ (1/2/3/4/5)	413(55.89)/201(65.05)/ 250(60.10)/102(67.55)/138(69.70)	326(44.11)/108(34.95)/ 166(39.90)/49(32.45)/60(30.30)	19.385	0.001
Surrounding choices-fertility influence^†^ (1/2/3/4/5)	450(55.90)/210(64.42)/ 247(61.29)/88(71.54)/109(69.87)	355(44.10)/116(35.58)/ 156(38.71)/35(28.46)/47(30.13)	21.294	<0.001

^†^These items were rated on a 5-point Likert scale from 1 (No influence) to 5 (Extremely great influence).

### 2.5 Statistical analysis

Valid questionnaire data collected in this study were imported into Stata 14.1 statistical software to establish a database, followed by data cleaning, sorting and analysis. For quantitative data, if normally distributed, they were described as mean ± standard deviation; otherwise, the median (interquartile range) was reported. Qualitative data were presented as frequency (n) and percentages (%). For quantitative data with normal distribution and homogeneous variances, the two-sample independent t-test (for two-group comparisons) or one-way analysis of variance (ANOVA; for multiple-group comparisons) was used for intergroup comparisons; otherwise, the rank-sum test was applied. The chi-square (χ^2^) test was used to compare intergroup differences in qualitative data, so as to explore potential factors associated with knowledge of the statutory fertility provision. Binary logistic regression analysis was employed to identify factors independently associated with knowledge of the statutory fertility provision among reproductive-aged women. Meanwhile, the variance inflation factor (VIF) was used to test for multicollinearity among independent variables and evaluate the rationality of model fitting. All statistical analyses were performed using Stata 14.1 software with two-tailed tests. A value of *p* < 0.05 was considered statistically significant.

To assess potential clustering effects arising from the multi-stage sampling design, the intraclass correlation coefficient (ICC) was calculated. The estimated ICC was 0.0262 (95% CI: 0.0001–0.0560), well below the conventional threshold of 0.05, indicating negligible clustering effects. Therefore, standard logistic regression without additional clustering adjustment was deemed appropriate.

Due to the small sample size of the ‘Widowed/Divorced’ category in marital status (*n* = 22, 0.91%), this category was merged with ‘Married and Childless’ (*n* = 206) to form a combined ‘Married and Childless / Widowed / Divorced’ group (*n* = 228). This merging was based on the shared characteristic of being currently without children.

Despite this merging, in the full multivariate model, the ‘Married and Childbearing’ category was automatically dropped due to quasi-complete separation—a statistical phenomenon where a category is fully predicted by the combination of other covariates. This is not a data quality issue; all three marital status categories had sufficient observations (*n* = 587, 228, and 1,607, respectively), and no multicollinearity was detected (all VIF < 5). The ‘Unmarried and Childless’ category served as the reference group. This limitation is acknowledged in the Discussion section.

### 2.6 Quality control

Firstly, participants were recruited via a random sampling approach to control selection bias to the greatest extent possible. Secondly, extensive publicity efforts were implemented to improve participant engagement, which served to reduce nonresponse rates and biases arising from subjective reporting errors.

### 2.7 Ethics approval

The procedures followed by the Institute are in line with the ethical standards of the Declaration of Helsinki (1964, most recently revised in 2013) established by the World Medical Association. And this study was approved by the Ethics Review Committee of Shandong Second Medical University (No. 2023YX-139).

## 3 Results

A total of 2,700 questionnaires were distributed, and 2,422 valid responses were collected (effective response rate: 89.70%). Among the respondents, 56.69% had a vague understanding of the statutory fertility provision, whereas 43.31% possessed an adequate level of knowledge of this provision.

### 3.1 Univariate analysis of factors associated with knowledge of the statutory fertility provision among reproductive-aged women

Five categories of variables, including more than 20 items—personal factors (residence, age, educational background, marital status, original family, occupation), economic status (monthly total family income / expenditure), traditional concepts (influence of the concept of “continuing the family line,” influence of the concept of “more sons, more happiness,” influence of the concept of “raising sons for old age security”), fertility intention (expected age at first birth, post-marital fertility time preference, preferred number of children / actual), and surrounding expectations (influences of social expectations and surrounding people’s choices)—were all significantly associated with knowledge of the statutory fertility provision (all *p* < 0.05; see Table 1).

### 3.2 Logistic regression analysis of factors associated with knowledge of the statutory fertility provision among reproductive-aged women

#### 3.2.1 Collinearity test of independent variables

The variance inflation factor (VIF) values of all independent variables were less than 10, with a mean VIF of 3.03, which was far below the critical threshold for severe multicollinearity (VIF > 10). These findings indicated no severe multicollinearity among the independent variables in the model (Table 2). This result meets the requirements for multicollinearity in logistic regression analysis.

**Table 2:** Multivariate logistic regression analysis of factors associated with knowledge of the statutory fertility provision.

Variables	Subgroups	*β*	*p* value	OR	95%CI for OR	
					Lower	Upper
Residence	Urban*	Reference				
	Rural	−0.394	0.024	0.675	0.480	0.949
Age (years old)	18–27*	Reference				
	28–37	−0.267	0.248	0.766	0.487	1.204
	38–45	−0.390	0.102	0.677	0.424	1.080
Educational background	Junior High School or Below*	Reference				
	Senior High School/Vocational High School/Technical Secondary School	0.096	0.687	1.101	0.689	1.761
	Undergraduate/College	0.413	0.091	1.511	0.936	2.441
	Postgraduate or Above	0.425	0.166	1.530	0.839	2.792
Marital status	Unmarried and Childless*	Reference				
	Married and Childbearing	0.014	0.940	1.014	0.709	1.449
Original family	Nuclear Family*	Reference				
	Stem Family	−0.125	0.255	0.882	0.711	1.095
	Joint Family	−0.100	0.685	0.904	0.557	1.469
	Single-Parent Family	0.586	0.212	1.797	0.715	4.513
	Reconstituted Family	0.128	0.756	1.137	0.507	2.548
Occupation	Public Service and Enterprises/Institutions*	Reference				
	Private and Individual Operation	0.016	0.929	1.017	0.708	1.459
	Physical Labor and Agricultural Work	0.433	0.059	1.542	0.983	2.418
	Flexible and Unfixed Occupation	−0.108	0.498	0.898	0.657	1.226
	Unemployed	−0.122	0.680	0.885	0.497	1.578
Monthly total family income (Yuan)	<4000*	Reference				
	4,001–8,000	0.050	0.783	1.051	0.737	1.499
	8,001–15,000	0.170	0.401	1.186	0.796	1.766
	>15,001	0.175	0.469	1.192	0.741	1.916
Monthly total family expenditure (Yuan)	<4000*	Reference				
	4,001–8,000	0.295	0.028	1.343	1.032	1.748
	8,001–15,000	0.144	0.430	1.155	0.808	1.651
	>15,001	0.290	0.427	1.336	0.653	2.734
Influence of the concept of “continuing the family line”	No Influence*	Reference				
	Little Influence	−0.245	0.292	0.782	0.496	1.235
	Moderate Influence	0.220	0.437	1.246	0.716	2.168
	Great Influence	0.087	0.821	1.091	0.513	2.318
	Extremely Great Influence	−0.177	0.757	0.838	0.273	2.574
Influence of the concept of “more sons, more happiness”	No Influence*	Reference				
	Little Influence	0.162	0.479	1.176	0.751	1.840
	Moderate Influence	0.014	0.957	1.104	0.612	1.680
	Great Influence	−0.026	0.940	0.975	0.502	1.893
	Extremely Great Influence	0.133	0.800	1.142	0.408	3.194
Influence of the concept of “raising sons for old age security”	No Influence*	Reference				
	Little Influence	0.184	0.385	1.201	0.794	1.818
	Moderate Influence	0.061	0.794	1.063	0.674	1.676
	Great Influence	−0.170	0.586	0.843	0.457	1.557
	Extremely Great Influence	0.131	0.775	1.140	0.465	2.795
Expected age at first birth (years old)	20–23*	Reference				
	24–27	0.298	0.158	1.348	0.891	2.039
	28–30	0.552	0.018	1.736	1.101	2.737
	31–34	0.847	0.005	2.332	1.288	4.221
	>35	−0.079	0.895	0.924	0.286	2.989
Post-marital fertility preference	Same Year*	Reference				
	1–2 Years Later	0.219	0.417	1.245	0.733	2.117
	3–4 Years Later	0.355	0.235	1.426	0.794	2.562
	More Than 5 Years Later	0.232	0.652	1.261	0.461	3.448
	Let Nature Take Its Course	0.301	0.310	1.351	0.756	2.415
Preferred number of children expected	0*	Reference				
	2	0.271	0.061	1.311	0.987	1.740
	3	1.159	<0.001	3.187	1.891	5.369
	>3	0.740	0.013	2.097	1.165	3.772
Actual-scenario fertility expectation	0*	Reference				
	1	0.471	0.120	1.602	0.885	2.900
	2	0.249	0.419	1.282	0.701	2.345
	3	0.609	0.151	1.839	0.800	4.227
	>3	0.289	0.686	1.334	0.330	5.389
Social expectation-fertility influence	No Influence*	Reference				
	Little Influence	−0.124	0.575	0.883	0.572	1.364
	Moderate Influence	0.090	0.706	1.093	0.687	1.742
	Great Influence	0.063	0.843	1.065	0.573	1.978
	Extremely Great Influence	−0.262	0.439	0.770	0.396	1.493
Surrounding choices-fertility influence	No Influence*	Reference				
	Little Influence	−0.360	0.097	0.698	0.456	1.067
	Moderate Influence	−0.362	0.121	0.696	0.440	1.100
	Great Influence	−0.640	0.069	0.527	0.264	1.052
	Extremely Great Influence	−0.316	0.427	0.759	0.334	1.591

*Indicates the reference group. The ‘Widowed / Divorced’ category was merged with ‘Married and Childless’ due to small sample size (*n* = 22). The ‘Married and Childbearing’ category was automatically dropped due to quasi-complete separation in the full model.

#### 3.2.2 Binary logistic regression analysis

Results showed that residence, monthly total family expenditure, expected age at first birth, and preferred number of children were independently associated with knowledge of the statutory fertility provision among reproductive-aged women (all *p* < 0.05).

Rural women had a significantly lower likelihood of having adequate knowledge of this provision than urban women (OR = 0.675, 95% CI: 0.480–0.949, *p* = 0.024).

Participants with 4,001–8,000 Yuan monthly total family expenditure had a significantly higher probability of having adequate knowledge of this provision than those with <4,000 Yuan monthly total family expenditure (OR = 1.343, 95% CI: 1.032–1.748, *p* = 0.028).

Compared with participants whose expected age at first birth was 20–23 years, those with an expected age of 28–30 and 31–34 years had 1.736 and 2.332 times higher odds of having adequate knowledge of this provision, respectively (OR = 1.736, 95% CI: 1.101–2.737, *p* = 0.018; OR = 2.332, 95% CI: 1.288–4.221, *p* = 0.005).

Compared with participants whose preferred number of children was 0, those whose preferred number was 3 and more than 3 had 3.187 and 2.097 times higher probability of having adequate knowledge of this provision, respectively (OR = 3.187, 95% CI: 1.891–5.369, *p* < 0.001; OR = 2.097, 95% CI: 1.165–3.772, *p* = 0.013).

Detailed data were presented in Table 2.

## 4 Discussion

This study aimed to identify factors associated with knowledge of the statutory fertility provision among reproductive-aged women in Shandong Province.

The findings revealed a substantial knowledge gap, with more than half of the target population lacking sufficient understanding of this fundamental policy rule. Residence, monthly total family expenditure, expected age at first birth, and preferred number of children were identified as key associated factors. Such a low level of policy awareness may directly undermine policy effectiveness, as previous research has clearly indicated that insufficient awareness of fertility policies is a key factor constraining fertility intentions (31). This level of awareness is consistent with the problem of general unawareness identified in existing studies (32). Given that Shandong Province is a highly populous province, such a low rate of policy awareness among its reproductive-aged women may have important implications for regional fertility levels (33, 34). These findings indicate the need for more effective, targeted, and accessible policy communication strategies (15).

### 4.1 Residence

According to the current study, place of residence is significantly associated with knowledge of the statutory fertility provision, with rural residents having a lower level of such knowledge than urban residents. This finding is consistent with previous studies (17, 35). Several factors may contribute to this urban–rural disparity. Firstly, urban women generally have higher educational attainment than rural women in China (36), and higher education is associated with stronger cognitive and comprehension skills (37, 38). Thus, urban women may have a greater capacity to understand policy information. Secondly, the availability and accessibility of grassroots government services—such as family planning offices and community service centers—are better in urban areas than in rural areas (39). In this context, policy dissemination is far more common and accessible in urban settings. These factors may help explain the observed urban–rural difference in knowledge of the statutory fertility provision.

### 4.2 Monthly total family expenditure

The results revealed that monthly total family expenditure may be associated with adequate knowledge of the statutory fertility provision. Only the group with a monthly expenditure of 4,001–8,000 Yuan showed a statistically significant difference and was positively associated with adequate knowledge of this provision, whereas no significant association was observed in other expenditure groups. These findings indicate that the relationship between family expenditure and knowledge of this provision is not a simple linear correlation. This finding aligns with previous research (40). Several explanations may account for this non-linear pattern. Firstly, economic resources may influence access to policy information (37). Women in the middle-expenditure group may have more stable economic conditions and more diverse information channels, while those with lower expenditure may face greater constraints in accessing policy-related information (41). Secondly, women in the middle-expenditure group may be at a life stage where fertility planning is particularly salient, which could increase their attention to relevant policies (40). In contrast, women with higher expenditure may have competing demands on their time and attention, potentially reducing their focus on fertility policy information (42, 43). Thirdly, the match between individual fertility intentions and policy provisions may influence awareness of the statutory fertility limit. Women whose fertility desires align with current policies may have a more direct personal interest in understanding the statutory fertility provisions, potentially contributing to the non-linear association observed in this study (40, 44, 45).

### 4.3 Expected age at first birth

Expected age at first birth emerged as a factor associated with adequate knowledge of the statutory fertility provision in this study. Women who planned to have their first birth at 28–30 years old and 31–34 years old showed significantly higher knowledge levels than those planning at 20–23 years old (OR = 1.736 and 2.332, respectively, both *p* < 0.05). This finding corroborates the conclusions of prior investigations (46). This difference may be attributable to several factors. Firstly, women who plan to have their first birth at 28–34 years old may have clearer and more specific fertility timelines. Such clear planning may make fertility policies more relevant to their personal circumstances, potentially increasing their attention to policy information (47). In contrast, women who plan their first birth at 20–23 years old may have less defined fertility plans and may perceive less personal relevance, which could be associated with lower levels of policy awareness (48). Secondly, women planning their first birth at 20–23 years old are often in a life stage focused on education or early career development, where fertility policies may not be a primary concern (49, 50). By contrast, women planning their first birth at 28–34 years old may be more directly affected by fertility policies, as they are approaching or within the typical childbearing age range, which may increase their attention to relevant policy information (51).

### 4.4 Preferred number of children

According to the present study, preferred number of children may be identified as a factor associated with adequate knowledge of the statutory fertility provision. Women who preferred 3 children and more than 3 children showed significantly higher knowledge levels than those preferring 0 children (OR = 3.187 and 2.097, respectively, both *p* < 0.05). This result is consistent with previous studies (52). The observed difference in knowledge levels between women who preferred 3 or more children and those who preferred 0 children may be related to variations in fertility intention (53). Women who prefer three or more children generally have stronger fertility intentions and more concrete fertility plans, which may create more pressing practical demands for supporting policies such as childcare subsidies, nursery services, and maternity leave protection (54). Such demands may motivate active information-seeking, enabling them to develop a more comprehensive understanding of policy details (55). In contrast, women with low fertility intention or no fertility plans may lack relevant policy application scenarios, may pay less attention to fertility policies, and may invest less effort in understanding them, thus potentially making it more difficult to achieve adequate policy awareness (56).

TPB is traditionally applied to intentional behaviors. However, knowledge acquisition can itself be viewed as a behavior—individuals must actively seek out, process, and retain policy information. In this sense, the TPB constructs may be interpreted as factors associated with the motivation to acquire policy knowledge, which subsequently affects actual knowledge levels. Within this extended framework, the observed associations provide meaningful insights. The significant positive association between fertility intention and knowledge of the statutory fertility provision is consistent with TPB’s core premise—individuals with stronger fertility intentions may be more motivated to seek out information about the statutory fertility provision relevant to their reproductive plans (30). However, the non-significant results for traditional fertility concepts and social expectations in the adjusted model are also interpretable within the TPB framework. As demonstrated by Armitage and Conner (30), the effects of attitudes and subjective norms on behavior are largely mediated by intentions rather than direct. This suggests that cultural norms and social pressures may influence knowledge of the statutory fertility provision primarily through fertility intentions, rather than exerting direct effects.

Similarly, the non-significant results for education, income, and occupation in the adjusted model may reflect that the association between socioeconomic status and knowledge of the statutory fertility provision is attenuated when fertility intentions are accounted for. Individuals with higher socioeconomic status may have better access to information in general, but this advantage may not translate into higher knowledge of the statutory fertility provision unless they have relevant fertility intentions.

## 5 Study strengths, limitations and policy implications

This study employed a multistage stratified cluster sampling method and conducted reliability and validity testing on the questionnaire to ensure data quality. Firstly, the sampling method was scientifically rigorous, which ensured strong representativeness of the sample. Secondly, the effective sample size was 2,422, providing adequate statistical power. Thirdly, the questionnaire demonstrated good reliability and validity, with a KMO value of 0.940, a significant Bartlett’s test of sphericity (*p* < 0.001), and a Cronbach’s alpha coefficient of 0.955.

However, several limitations should be acknowledged. Firstly, the study employed a cross-sectional design, which can only reveal associations between variables and cannot establish causal relationships or temporal sequences. Secondly, the study only included reproductive-aged women with household registration in Shandong Province, which limits the generalizability of the findings to other geographic contexts. Thirdly, the data were collected through self-administered questionnaires, which are subject to self-report biases, including potential recall bias and social desirability bias. Fourthly, this study assessed knowledge of the statutory fertility provision using a single-item question. It should be noted that this single-item measure does not capture the full spectrum of fertility policy awareness, which ideally encompasses knowledge of multiple supportive policy dimensions, including maternity leave entitlements, childcare subsidies, eligibility criteria, and policy access procedures. Consequently, the findings should be interpreted as reflecting knowledge of the core statutory rule rather than comprehensive fertility policy awareness. Future research should develop and validate multi-dimensional instruments that cover the full range of fertility-support policies to enable a more complete assessment of policy awareness. Fifthly, although a multi-stage sampling design was employed, potential clustering effects, design effects, and sampling weights were not explicitly accounted for in the statistical analyses. This may affect the precision of the estimates. Future studies could benefit from incorporating sampling weights and accounting for design effects in the statistical analyses. Sixthly, due to quasi-complete separation in the full multivariate model, the ‘Married and Childbearing’ category of the marital status variable could not be independently estimated and was automatically dropped by the statistical software. This prevented a full examination of the association between marital status and knowledge of the statutory fertility provision. However, the retained categories still provide meaningful information, and the remaining core findings remain valid. Future studies with larger samples or alternative coding strategies may further explore the role of marital status. Seventhly, applying the Theory of Planned Behavior (TPB) to a cognitive outcome (policy knowledge) rather than a behavioral outcome represents a theoretical extension of the original framework. While this extension may provide useful insights into the determinants of policy knowledge acquisition, future research may benefit from complementary frameworks more specifically designed to explain knowledge and information processing, such as the Health Belief Model or the Information Seeking Model. In addition, other methodological improvements—such as longitudinal designs, multi-regional sampling, and objective behavioral indicators—are recommended to address the remaining limitations of this study.

## 6 Conclusion

This study found that only 43.31% of reproductive-aged women had clear knowledge of the statutory fertility provision. The results showed that the main factors associated with knowledge of the statutory fertility provision included residence, monthly total family expenditure, expected age at first birth, and preferred number of children. Based on these findings, the publicity and popularization of fertility policies should be optimized in multiple ways. Differentiated communication strategies should be adopted for urban and rural residents to expand access to policy information. Policy interpretation should be tailored to families with different economic levels. In addition, targeted publicity should be strengthened among women with earlier fertility plans and weaker fertility intentions, so as to improve their attention and understanding of fertility support policies.

Future research could expand the study area to conduct multi-province comparative analyses, explore the dynamic changes in knowledge of the statutory fertility provision and their impact on actual fertility behavior. Qualitative studies may also be combined to further analyze barriers to information acquisition, so as to provide a scientific basis for formulating more targeted fertility support policies.

## Data Availability

The raw data supporting the conclusions of this article will be made available by the authors, without undue reservation.
